# Automated alignment of XFEL nanofocusing mirrors via wavefront optimization

**DOI:** 10.1107/S1600577525008434

**Published:** 2025-10-24

**Authors:** Jumpei Yamada, Gota Yamaguchi, Ichiro Inoue, Taito Osaka, Yuichi Inubushi, Makina Yabashi

**Affiliations:** a Research Center for Precision Engineering, Graduate School of Engineering Osaka University 2-1 Yamada-oka Suita Osaka565-0871 Japan; b RIKEN SPring-8 Center, 1-1-1 Kouto, Sayo, Hyogo679-5148, Japan; c Japan Synchrotron Radiation Research Institute 1-1-1 Kouto Sayo Hyogo679-5198 Japan; SLAC National Accelerator Laboratory, USA

**Keywords:** X-ray free-electron lasers, X-ray nanofocusing, X-ray mirrors, wavefront sensing, beamline automation

## Abstract

An automated alignment procedure, based on wavefront measurement with a single-grating interferometer, has been developed for precise tuning of Kirkpatrick–Baez nanofocusing mirrors for X-ray free-electron lasers (XFELs). This approach optimizes focus size and maximizes peak intensity while minimizing aberrations. Wavefront errors are quantitatively correlated with alignment deviations – incidence angle, perpendicularity and astigmatism – via Legendre polynomial analysis. These errors are subsequently corrected through a straightforward optimization process. Implemented at the SPring-8 Angstrom Compact Free-Electron Laser (SACLA), the system consistently achieves a reproducible XFEL focus below 150 nm × 200 nm within 10 min. Routine operation at SACLA demonstrates the reliability and efficacy of this method, enabling rapid restoration of optimal nanofocusing conditions.

## Abstract 2

An automated alignment system for nanofocusing Kirkpatrick–Baez mirrors has been developed at SACLA. A wavefront optimization technique routinely provides nanofocused XFEL beams.

## 1. Introduction

X-ray free-electron lasers (XFELs) are new pulsed light sources with unprecedentedly high brightness and ultra-short pulse duration. By tightly focusing XFEL pulses, one can enhance the peak intensity and produce exotic states in matter by X-ray irradiation. Currently, focusing an XFEL beam down to a spot size of several tens of nanometres with intensity up to 10^20^ W cm^−2^ has been demonstrated (Yumoto *et al.*, 2013 ▸; Mimura *et al.*, 2014 ▸; Yumoto *et al.*, 2020 ▸; Seaberg *et al.*, 2022 ▸; Kim *et al.*, 2025 ▸), which is sufficient for the exploration of nonlinear optical phenomena (Yoneda *et al.*, 2014 ▸; Yoneda *et al.*, 2015 ▸; Tamasaku *et al.*, 2014 ▸; Fuchs *et al.*, 2015 ▸) and their applications (Tamasaku *et al.*, 2018 ▸; Kroll *et al.*, 2018 ▸; Tamasaku *et al.*, 2023 ▸).

Among various types of X-ray optical devices, total-reflection X-ray mirrors have been extensively employed for the nanofocusing of XFELs. A total-reflection mirror with Kirkpatrick–Baez (KB) geometry (Kirkpatrick & Baez, 1948 ▸), which consists of two grazing-incidence elliptical mirrors arranged perpendicularly to each other, can achieve a nanometre-scale XFEL focusing spot size with high throughput, wide-range achromaticity and high radiation hardness. However, nanofocusing KB mirrors exhibit stringent tolerances for incident angle errors because the elliptical mirror cannot satisfy the Abbé sine condition. This limitation necessitates time-consuming optics alignment requiring a lot of trial and error. Additionally, the focusing condition is sensitive to temperature changes, requiring frequent optical re-tuning that reduces experimental efficiency. Another difficulty lies in the diagnostic method of alignment errors of KB mirrors in XFELs. While a Foucault testing method and a knife-edge scanning method have been adopted, their application to alignments of nanofocusing KB mirrors for XFELs is hindered by pointing jitter in incoming XFEL pulses and ablation damage to knife edges caused by high intensities at the focal position. Moreover, the Foucault method is subjective, and the knife-edge method measures only the beam size; neither provides quantitative alignment error information for KB mirrors.

In this paper, we report an automated alignment system for XFEL nanofocusing mirrors based on wavefront sensing. A single-grating interferometer (s-GI) with negligible systematic errors (Yamada *et al.*, 2020 ▸) was employed as a wavefront sensor. Wavefront errors were quantitatively correlated with alignment deviations using Legendre polynomial analysis. The rapid and reliable measurements provided by the s-GI enabled precise nanofocus optimization through an automated alignment procedure. This system has been implemented at the SPring-8 Angstrom Compact Free-Electron Laser (SACLA), achieving a reproducible XFEL focus below 150 nm × 200 nm.

## 2. Methods

**Figure 1:**
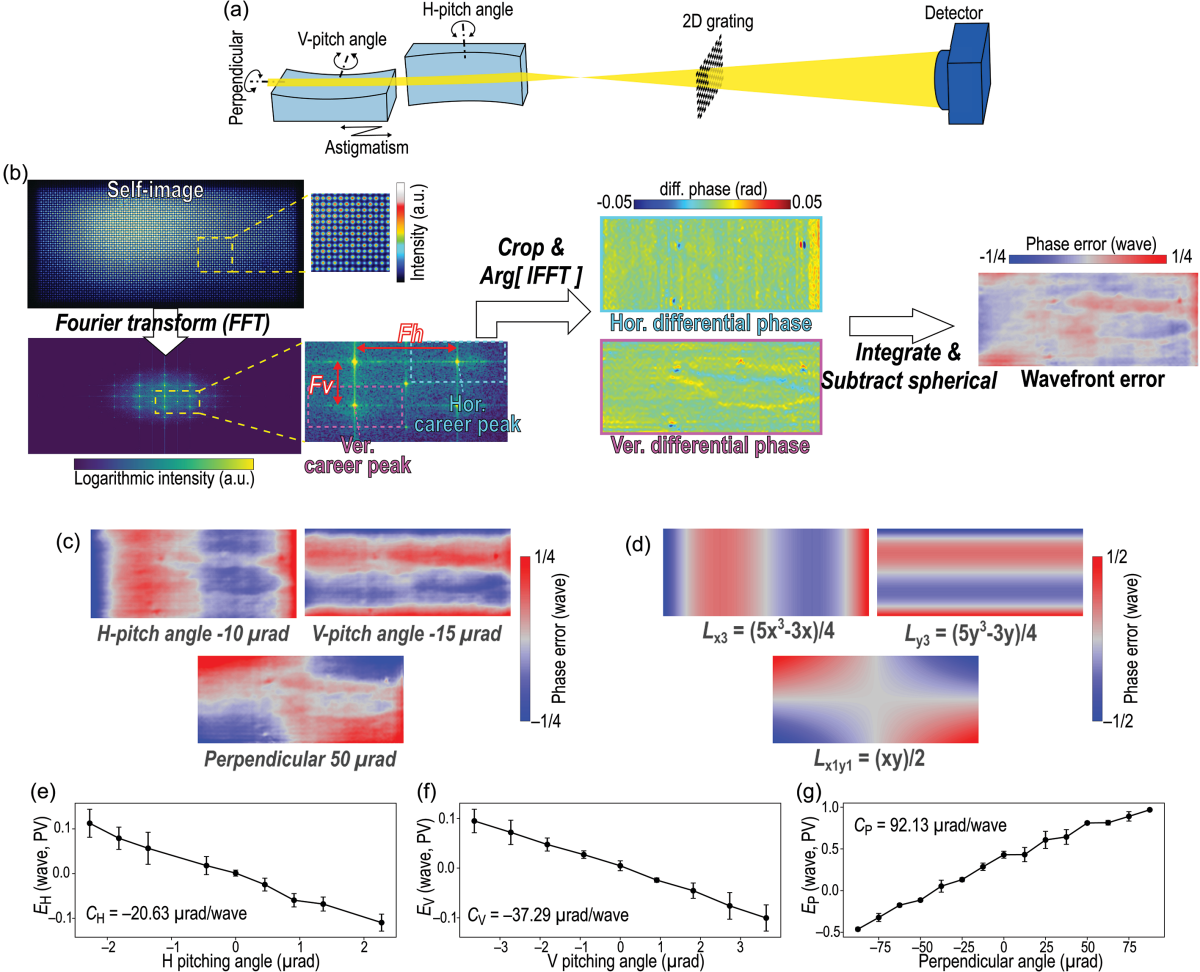
(*a*) Schematic illustration of the KB nanofocusing mirrors and a single-grating interferometer equipped in EH5 on BL3 at SACLA. (*b*) Workflow for calculating the wavefront error from the acquired self-image, based on the Fourier transform method. The abbreviations ‘Ver.’ and ‘Hor.’ indicate the vertical and horizontal directions, respectively. (*c*) Wavefront errors with purposely induced alignment errors. (*d*) Calculated profiles of the Legendre polynomials in equations (2)–(4). (*e*)–(*g*) Obtained relationships between the fitted amplitudes of wavefront errors (*E*_H_, *E*_V_ and *E*_P_) and corresponding angular misalignments for (*e*) horizontal pitching angle, (*f*) vertical pitching angle and (*g*) perpendicularity.

Fig. 1 ▸(*a*) shows a schematic of the optical setup. The parameters of the nanofocusing KB mirrors are as follows: numerical aperture of 2.0 × 10^−3^ in the horizontal (H) direction and 1.0 × 10^−3^ in the vertical (V) direction, typical focusing spot size of 120 nm (H) and 200 nm (V) at photon energies of 5–12 keV, and mirror lengths of 250 mm. Further details are reported elsewhere (Yumoto *et al.*, 2020 ▸). The s-GI (Yamada *et al.*, 2020 ▸) utilizes a low-distortion X-ray detector and two-dimensional (2D) checker-board π-phase gratings made of tantalum (NTT Advanced Technology Co.). The grating period *p*_0_ is primarily 6.4 µm for photon energies of 6.5–10.0 keV and Talbot order *m* of 3/8, with additional gratings of periods 4.4 µm (10.0–12.0 keV, *m* = 3/8 or 5/8) and 7.0 µm (4.0–6.5 keV, *m* = 3/8) for broader energy ranges. Although the phase shift generated by the grating theoretically changes depending on the photon energy, especially across the energy range between 6.5 and 10 keV, this effect provides slight visibility changes at the distance with a fractional Talbot order of 3/8. The focus–grating distance *f* is set to be around 100–150 mm, and the focus–detector distance *L* is 1–2 m, ensuring compatibility with XFEL experiments using the KB nanofocusing mirror system in EH5 on BL3 at SACLA (Tono *et al.*, 2013 ▸). This s-GI is not permanently integrated into the focusing system but can be optionally incorporated into experiments when the setup conditions are satisfied. The necessary configuration adjustments can be implemented in a straightforward and technically feasible manner. In this work, the s-GI is applied to a divergent beam, where the small-angle approximation is sufficiently valid. The effect of measurement error caused by the beam divergence, as discussed by Yamada *et al.* (2020 ▸), is less than λ/700 and therefore negligible.

The wavefront is evaluated using Talbot interference fringes of the grating (termed a self-image). Fig. 1 ▸(*b*) illustrates the computation workflow to obtain the wavefront errors. The radius of curvature of the wavefront *R*, which quantifies the astigmatism error, is given by
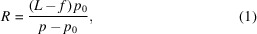
where *p* denotes the measured period of the self-image. Specifically, *p* is derived from the number of acquired fringes, which is equivalent to the position of the adjacent spectrum to the zero-order peak in reciprocal space [labelled *Fh* and *Fv* in Fig. 1 ▸(*b*)]. *R* is calculated for both the H and V directions, and the astigmatism error *E*_A_ is defined as their difference. The wavefront slope maps, corresponding to the differential of the wavefront along the V and H directions, are reconstructed using the Fourier transform method (Takeda *et al.*, 1982 ▸). The wavefront profile is then obtained through 2D integration, for which the cosine transform integration (Bon *et al.*, 2012 ▸) is employed in this study. This approach, relying on several Fourier transform operations, is computationally efficient, enabling rapid wavefront reconstruction. To compute the wavefront error, quadratic terms in the wavefront corresponding to the astigmatism error in the H and V directions are subtracted.

As previous research suggested (Mercère *et al.*, 2006 ▸; Merthe *et al.*, 2012 ▸; Zhou *et al.*, 2018 ▸; Kahnt *et al.*, 2022 ▸), the wavefront error works as a diagnostic tool for alignment errors of KB mirrors. While Zernike polynomials are commonly used for wavefront aberration analysis in optics with circular-aperture and rotationally symmetric lenses (He *et al.*, 2010 ▸), they are not optimal for rectangular-aperture KB mirrors, which introduce orthogonally independent aberration components. Therefore, we adopted normalized Legendre polynomials for the analysis. The peak-to-valley (PV) amplitudes of specific wavefront components were correlated with alignment deviations, namely the pitching angle error of the H mirror *E*_H_, the pitching angle error of the V mirror *E*_V_ and the perpendicularity error of the KB mirror *E*_P_, by least-squares fitting to the following equations,
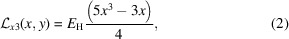

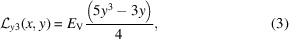

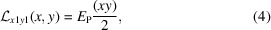
where 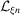 indicates the *n*th-order Legendre polynomial along the ξ (*x* or *y*) direction. Here, *x* and *y* denote the normalized spatial coordinates (*e.g.* relative pixel size) along the horizontal and vertical directions, respectively, ranging from −1 to 1. The agreement between the wavefront errors with deliberately induced misalignments [Fig. 1 ▸(*c*)] and the corresponding fitted Legendre polynomial components [Fig. 1 ▸(*d*)] indicates the validity of this evaluation method. The other alignment axes, such as in-plane rotation, have a large tolerance to be compatible with simple off-line tuning.

**Figure 2:**
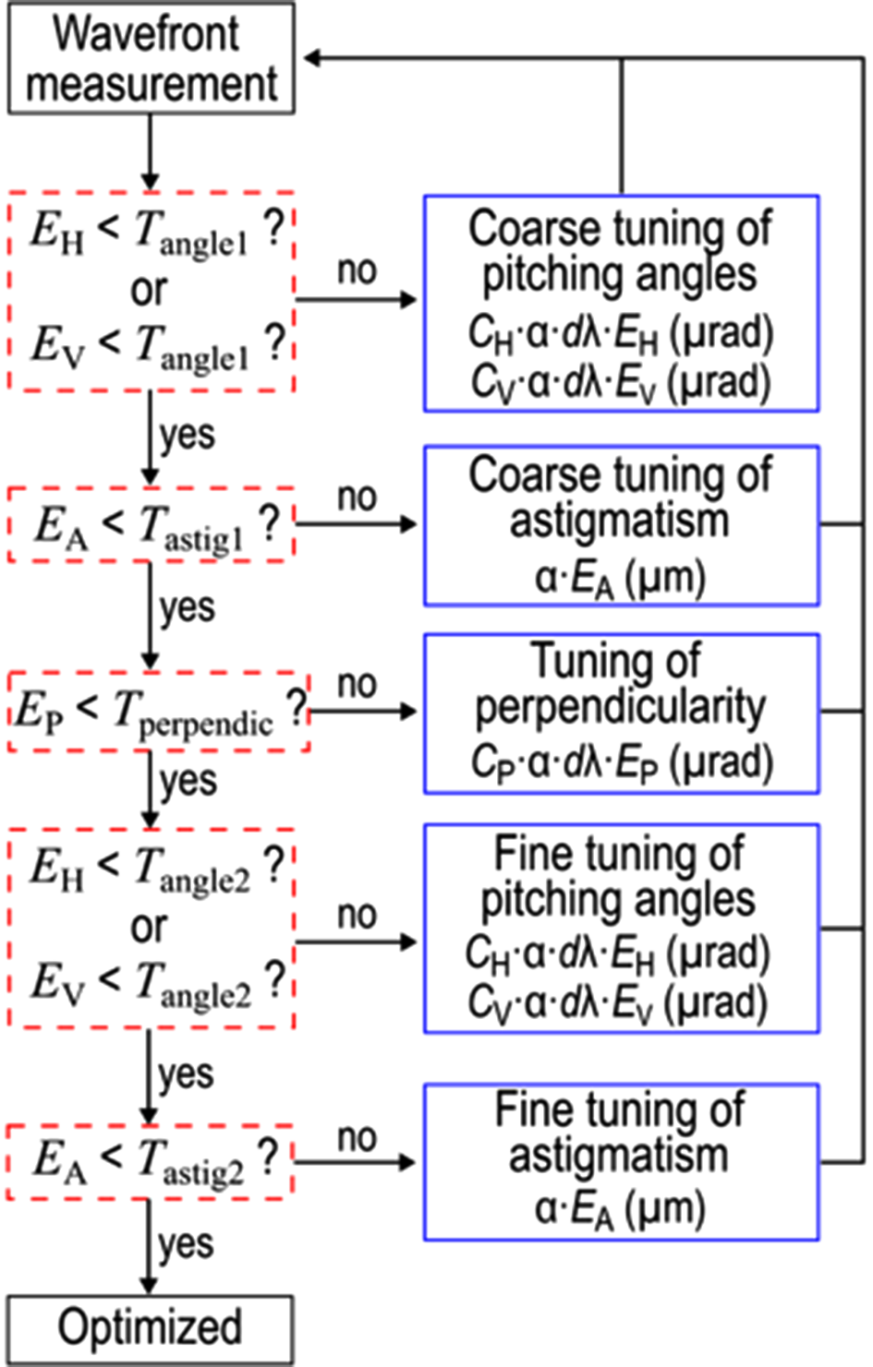
Diagram of the wavefront optimization procedure.

Figs. 1 ▸(*e*)–1 ▸(*g*) show the experimentally obtained relationships between the fitted PV values (*E*_H_, *E*_V_ and *E*_P_) and their corresponding angular deviations at a photon energy of 9.1 keV. These results confirm clear linear dependencies, from which the sensitivity coefficients of *C*_H_, *C*_V_ and *C*_P_ were derived. These coefficients are subsequently used in the wavefront optimization procedure illustrated in Fig. 2 ▸. Given the high sensitivity of pitching angle adjustments, the optimization is performed in two stages, coarse and fine. Additionally, astigmatism adjustment follows the pitching angle tuning, as changes in the incidence angle induce shifts in the focal position. The required corrections for each alignment axis are calculated using the respective coefficients for each angular motion, incorporating a weight factor α for convergence. A wavelength-dependent correction factor *d*λ [*d*λ = λ (nm)/0.136] is also applied, since the coefficients (*C*_H_, *C*_V_ and *C*_P_) were derived at a photon energy of 9.1 keV. According to Rayleigh’s quarter-wavelength rule (Born & Wolf, 1999 ▸) and considering the depth of focus of approximately 60 µm, the typical thresholds for the tuning procedures *T*_angle1_, *T*_angle2_, *T*_astig1_, *T*_astig2_ and *T*_perpendic_ were set to λ/4, λ/10, 200 µm, 40 µm and λ/4, respectively. Adopting a weight factor of α = 0.8 ensured stable convergence, while further optimizations might be possible.

## 3. Results

**Figure 3:**
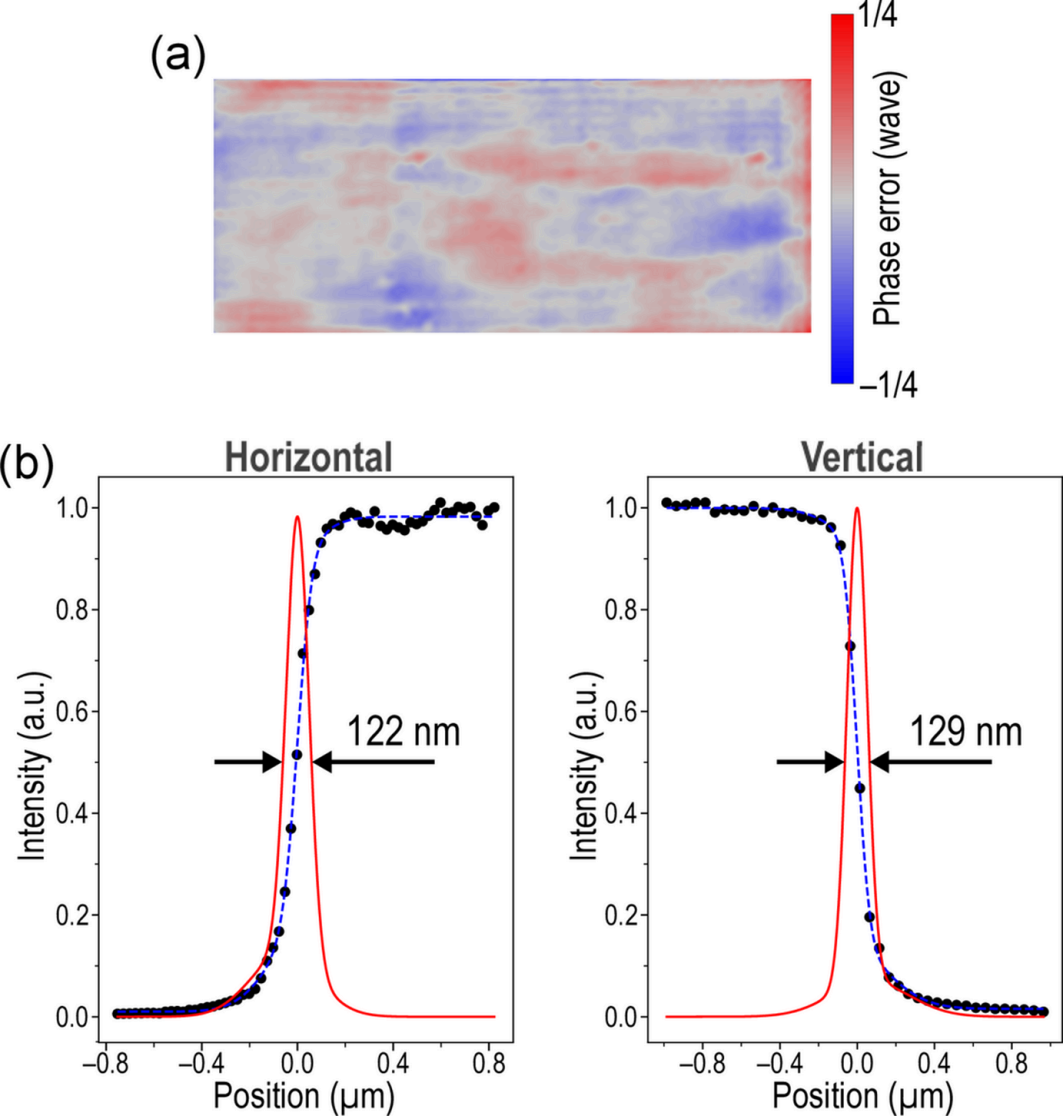
(*a*) Wavefront error profile after optimization. (*b*) Intensity distributions at focus characterized by the knife-edge scanning method.

Based on the aforementioned procedure, automated tuning of XFEL nanofocusing mirrors was performed at a photon energy of 9.1 keV. To mitigate ablation damage of the grating by the intense XFEL beam, the incident pulse energy was adequately attenuated with well polished silicon attenuators. The self-images for the wavefront reconstruction were obtained from 30 pulse averages, *i.e.* exposure times of approximately 1 s. Although five self-images were acquired for statistical robustness, the measurement and wavefront reconstruction process was completed in less than 10 s. Fig. 3 ▸(*a*) shows the optimized wavefront error, which achieved an accuracy of less than λ/30 in root mean-square (r.m.s.). This satisfies the Maréchal criterion of λ/14 r.m.s., indicating diffraction-limited focusing performance. The residual wavefront error stems from slight mirror imperfections, particulate contamination on optical components (mirrors, windows and gratings) and diffraction from beamline slit edges. Following optimization, intensity profiles of the focus were measured using a conventional knife-edge scanning method with a 200 µm diameter gold wire. The obtained focused beam profiles along the H and V directions are presented in Fig. 3 ▸(*b*). A focused beam size of 122 nm (H) × 129 nm (V) was achieved, demonstrating successful optimization. Notably, the knife-edge scan results are based on averaged data from ten pulses per point, suggesting that individual pulses may yield even smaller beam sizes.

The automated nanofocus tuning system has been successfully implemented for routine operation at SACLA. Table 1 ▸ summarizes the results of the focus sizes measured immediately after wavefront optimization across nine experiments with photon energies ranging from 5.9 to 10.5 keV. While the achieved focusing spot sizes varied depending on the XFEL source conditions, particularly the source position and size, the system consistently reproduced nanofocused XFEL beams that reached intensities of 10^19^–10^20^ W cm^−2^, corresponding to peak photon densities of 5 × 10^32^ to 1 × 10^33^ photons s^−1^ mm^−2^. These results validate the efficacy of quantifying mirror alignment deviations from wavefront errors combined with the optimization procedure developed here. In all experiments, the wavefront optimization procedure required only 3–10 min, enabling rapid restoration of the focusing condition and enhancing the statistical reliability of the experiments.

## 4. Conclusion

An alignment tuning system for KB nanofocusing mirrors, based on wavefront optimization, has been developed and applied to the XFEL beam at SACLA. The automated procedure achieved an optimized wavefront with an accuracy of λ/30 r.m.s. The reliability of the system was demonstrated through the consistent reproduction of nanofocused XFEL beam sizes across nine different experiments spanning a wide photon energy range.

One recent application of a nanofocused XFEL beam is X-ray stimulated emission (Yoneda *et al.*, 2015 ▸; Doyle *et al.*, 2023 ▸), which utilizes two-colour XFEL pulses (Hara *et al.*, 2013 ▸; Inoue *et al.*, 2020 ▸). The developed tuning system is applicable to such pulses if the intensity and attenuation ratios of the two pulses are carefully considered. The wavefront optimization procedure has been extended to other nano­focusing mirrors at SACLA (Yamada *et al.*, 2024 ▸) and holds potential for application in synchrotron radiation X-ray sources. Specifically, their use with synchrotron radiation will facilitate cross-calibration with a highly precise method such as ptychography, providing even higher absolute accuracy of the nanofocus tuning.

The rapid and quantitative alignment tuning of KB nanofocusing mirrors presented here promises to broaden the utility of X-ray beams across diverse scientific disciplines.

## Figures and Tables

**Table 1:** Achieved XFEL focus sizes using the automated alignment procedure developed here The foci were measured by the knife-edge scanning method.

Date (year, month)	Horizontal focus size (nm, FWHM)	Vertical focus size (nm, FWHM)	Photon energy (keV)
2020, May	122	129	9.1
2021, January	134	185	9.1
2021, June	130	176	10.5
2021, December	145	146	9.1
2022, February	111	160	9.5
2022, October	106	144	9.1
2022, November	123	138	5.9
2023, June	82	114	6.4
2024, March	87	171	9.1
